# Enhancement of Adiponectin Ameliorates Nonalcoholic Fatty Liver Disease via Inhibition of FoxO1 in Type I Diabetic Rats

**DOI:** 10.1155/2018/6254340

**Published:** 2018-08-16

**Authors:** Xiang Xie, Dan Yan, Haobo Li, Qiqi Zhu, Jun Li, Yong-ping Fang, Chi Wai Cheung, Michael G. Irwin, Zhengyuan Xia, Qingquan Lian

**Affiliations:** ^1^Department of Anesthesiology, The Second Affiliated Hospital and Yuying Children's Hospital, Wenzhou Medical University, Wenzhou, China; ^2^Department of Anesthesiology, The University of Hong Kong, Pok Fu Lam, Hong Kong; ^3^Department of General Surgery, Huizhou First Hospital, Huizhou, Guangdong, China

## Abstract

Nonalcoholic fatty liver disease (NAFLD) is a common liver disease which has been previously shown to be associated with type 2 diabetes mellitus (T2DM). Recent research has indicated that type 1 diabetes mellitus (T1DM) is also involved in the development of nonalcoholic fatty liver disease, whereas the underlying mechanisms are largely unknown. Forkhead box O1 (FoxO1) and adiponectin (APN) have been proposed to play an important role in the processes in NAFLD in T1DM. We herein investigated the effects of FoxO1 and APN on the development of NAFLD and the underlying mechanism in streptozotocin-induced T1DM. Serum liver enzymes AST, ALT, and triglyceride (TG) were determined by commercially available kits. Blood glucose levels were measured by the OneTouch Ultra glucose meter. Relevant protein expression was tested by Western blot analysis. Results showed that serum AST, ALT, and TG were all significantly increased in T1DM rats, which was ameliorated by application of APN or selective inhibition of FoxO1 with AS1842856. Moreover, APN and AS1842856 both decreased the expression of liver nuclear FoxO1 which was significantly increased in diabetic rats. However, the inhibition of FoxO1 did not alter the expression of APN and its receptors. We also found that Akt1 expression was significantly declined in diabetic rat which was restored by APN and moderately and significantly increased by FoxO1 inhibition. It is concluded that APN ameliorates NAFLD via inhibition of FoxO1 through Akt1/FoxO1 signaling pathway.

## 1. Introduction

Nonalcoholic fatty liver disease (NAFLD), diagnosed by fat deposition in the liver without viral hepatitis or substantial alcohol consumption, is the most prevalent chronic liver disease. NAFLD represents a spectrum of liver abnormalities starting from simple hepatic steatosis to severe manifestations, such as inflammations and liver injury known as nonalcoholic steatohepatitis (NASH), which could further progress to cirrhosis, hepatocellular carcinomas, and ultimately liver failure. It is not yet entirely understood how the pathogenesis of NAFLD develops; however, the liver fat accumulation and development of inflammation and fibrosis seem to be the most important contributing factors. Previous studies have indicated that the prevalence of NAFLD in patients that suffer obesity/type II diabetes, which has been estimated to be greater than 70% [1–3], is closely linked with type 2 diabetes mellitus (T2DM) and obesity in epidemiology and pathophysiology [4]. However, recently NAFLD has been associated with type 1 diabetes mellitus (T1DM) [5], in which obesity is not considered to play a significant pathogenic role. In another study, nearly 27.7% (204/736) T1DM patients had NAFLD [6], while little research has been focused on the relationship between T1DM and NAFLD and the underlying mechanism remains unknown.

The adipose tissue has long been considered as an energy storage organ. Recently, a variety of findings illustrated its important role as an active endocrine organ that synthesizes adipokines, including adiponectin (APN), leptin, resistin, and visfatin that are involved in the progression and pathogenesis of NAFLD [7, 8]. Particularly, APN is regarded as an antidiabetic, antiatherogenic, and anti-inflammatory adipokine. It is reported that APN level was inversely related with hepatic fat accumulation [9], and plasma APN significantly decreased in patients with NAFLD, particularly in NASH patients [10]. We, therefore, supposed that increasing APN level may alleviate the pathology of NASH. Two adiponectin receptors (AdipoRs) have been identified to regulate biological effects of adiponectin, namely, AdipoR1 and AdipoR2. AdipoR1 is commonly expressed in various tissues (in relatively large amount in skeletal muscle) and selectively activating AMP-activated kinase (AMPK) in the liver, whereas AdipoR2 primarily exists in the liver [11] and mediates insulin sensitivity by activation of PPAR*α*. Both AdipoR1 and AdipoR2 interact with globular and full-length adiponectin, mediating fatty acid oxidation and glucose uptake [12]. Recent study shows that prompting APN and inhibiting the activation and expression of forkhead box protein O1 (FoxO1) is associated with attenuation of hepatic steatosis in high-fat- and high-sugar-treated rats [13].

The transcription factor forkhead box O1 (FoxO1) is the key intracellular targets of insulin effect, contributing to the regulation of lipid and glucose metabolism [14]. After the binding of insulin to insulin receptor (IR), Akt is activated, subsequently phosphorylating and excluding FoxO1 proteins from the nucleus to the cytoplasm, thus dampening its effects to promote glucose production [15], both in isolated hepatocytes [16] and transgenic mouse models [17]. Activation of FoxO1 may lead to dysregulation of lipid and glucose metabolism whereas FoxO1 ablation improves lipogenesis and helps restore lipid metabolism homeostasis [14, 18, 19]. As a selective antagonist of FoxO1, AS1842856 reduces hepatic glucose production through inhibition of FoxO1 transactivation [20] and suppresses adipogenesis [21].

Several studies reported that FoxO1 interacts with Akt [22] and Sirtuin 1 (SIRT1) [23], affecting hepatic AdipoR1/R2 gene expression [24, 25]. Moreover, it has been indicated that FoxO1 impedes PPAR*γ* function [26] which has been illustrated to enhance adiponectin secretion [27], while recent research also reported that adiponectin could regulate FoxO1 expression via APN-AMPK-FOXO signal pathway [28]. However, the interaction between FoxO1 and APN in NAFLD is still unclear. We hypothesize that APN ameliorates liver function by FoxO1 inhibition in NAFLD via Akt1 pathway.

## 2. Method and Material

### 2.1. Induction of Diabetes

Male Sprague-Dawley rats (250 ± 10 g, 6–8 weeks) were obtained from the Shanghai Laboratory Animal Center (Shanghai, China) and the Laboratory Animal Service Center (The University of Hong Kong). All rats were housed and given free access to standard rat chow and water. The investigation conformed to the procedures described in the Guide for the Care and Use of Laboratory Animals published by the United States National Institutes of Health (NIH Publication number 85-23, revised 1996). The experimental protocol used in this study was approved by the Wenzhou Medical University Laboratory Animal Ethics Committee and the Committee for Use of Live Animals in Teaching and Research (CULATR) of the University of Hong Kong. Diabetes was induced by a single tail vein injection of streptozotocin (STZ) at the dose of 65 mg/kg bodyweight (Sigma-Aldrich, St. Louis, MO) in 0.1 M citrate buffer (pH 4.5) or citrate buffer alone as control under anaesthesia with a combination of ketamine 67.7 mg/kg bodyweight and xylazine 6.77 mg/kg bodyweight. After 72-hour injection, blood glucose was measured using the OneTouch Ultra glucose meter (LifeScan Inc., USA), and rats with blood glucose levels over 15 mM were considered diabetic.

### 2.2. Treatments with APN Adenovirus and FoxO1 Inhibition with AS Treatment

Control and diabetic rats (*n* = 6 per group) were either untreated (group C, group D) or diabetic rats treated with recombinant adenovirus expressing adiponectin (group APN, *n* = 6) or luciferase injected via tail vein for 1 week prior to tissue collection [29]. The increased expression level of adiponectin was confirmed by an enzyme-linked immunosorbent assay kit (AdipoGen Inc., Incheon, South Korea). The values of APN were expressed as micrograms per milliliter in plasma. Another group of diabetic rats (*n* = 6) were treated with a selective FoxO1 inhibitor AS1842856 (AS). AS1842856 has an IC50 of 0.033 mM to inhibit FoxO1 and can potently block FoxO1 at a final concentration of 0.05–1 mM without showing cytotoxicity [20, 30]. Rats were administrated intragastrically with AS (100 mg/kg every time) or the same dose of *β*-cyclodextrin as solvent control 2 times a day with a 12-hour interval for a duration of 8 days before the rats were terminated. All rats were terminated at 5 weeks after induction of diabetes. Our preliminary study and/or former study showed that luciferase and *β*-cyclodextrin did not affect target proteins in the liver/plasma [31]; the respective groups were not shown in the result parts.

### 2.3. Measurements of General Characteristics

At 5 weeks after the onset of diabetes, the rats' water intake and food consumption were recorded; rats were weighed and then euthanized following anaesthesia with an intraperitoneal injection of pentobarbital sodium (65 mg/kg). Blood samples were obtained from the carotid artery after an overnight fast of 8–10 h, and plasma was extracted and stored at −80°C until analyzed. Serum total aspartate aminotransferase (AST), alanine aminotransferase (ALT), and triglyceride (TG) were determined using commercially available kits (Stanbio Laboratory, TX, USA), respectively. Liver weight was recorded for calculating the liver weight/body weight. Blood glucose levels (mM) were measured every other day by using the OneTouch Ultra glucose meter (LifeScan Inc., USA). Plasma insulin levels were analyzed using the Ultrasensitive rat Insulin ELISA Kit (Stanbio Laboratory, TX, USA).

### 2.4. Nucleocytoplasmic Separation

Frozen liver tissues were suspended in a buffer that contained 10 mM Tris, pH 7.5, 1.5 mM MgCl_2_, 10 mM KCl, and 0.1% Triton X-100 and lysed by homogenization. Nuclei were recovered by microcentrifugation at 7500 rpm for 5 min. The supernatant that contained cytoplasmic and membrane protein was collected and stored at −80°C for Western blot analysis. Nuclear proteins were extracted at 4°C by gently resuspending the nuclei pellet in a buffer that contained 20 mM Tris, pH 7.5, 20% glycerol, 1.5 mM MgCl_2_, 420 mM NaCl, 0.2 mM EDTA, and 0.1% Triton X-100, followed by 1 h incubation at 4°C with occasional vortexing. After microcentrifugation at 13,000 revolutions/min for 15 min at 4°C, the supernatant that contained nuclear protein was collected.

### 2.5. Frozen Section and Staining

Fresh liver tissue was embedded in OCT compound and then stored at −80°C. After that, we cut 10 um thick sections and mounted these on gelatin-coated slides. These slides were stored at −80°C until needed. Before staining, we warmed slides at room temperature for 30–60 minutes and fix in ice-cold acetone as fixatives for 5–10 minutes, then air dry the slides for 30–60 minutes. After that, we washed the slides in PBS and proceed to standard staining procedure with hematoxylin and eosin (H&E) and Oil Red O (Sigma, USA).

### 2.6. Western Blotting

The protein concentration of liver lysates was quantified by bovine serum albumin and measured with the absorption of Coomassie brilliant blue in the spectrophotometer. Thereafter, the samples were frozen at −20°C for later use. Proteins were assessed by standard Western blotting as described [32]. Briefly, equal quantities of protein were separated by SDS-PAGE and transferred to polyvinylidene difluoride membranes (PVDF, Millipore, Bedford, MA, USA). The membranes were blocked in 5% nonfat dry milk diluted with Tris Buffered Saline Tween-20 (TBST) (in mmol/L: Tris-HCl 20, NaCl 150, pH 7.5, 0.1% Tween 20) at room temperature for 1 h and then probed with antibodies against FoxO1, p-FoxO1, APN, AdipoR1, Akt1, Akt2, SIRT1, acetyl-FoxO1, *β*-actin, GAPDH, and Histone 3 (Cell Signaling Technology, Beverly, MA, USA) at 4°C overnight. After extensive washing, the membranes were incubated with secondary horseradish peroxidase-conjugated anti-mouse or anti-rabbit antibodies (diluted 1 : 2000; Amersham Biosciences, UK). The immunoblots were visualized using an enhanced chemiluminescence detection system (Amersham Pharmacia Biotech, Uppsala, Sweden).

### 2.7. Statistics

All values are presented as means ± SEM. Comparisons between multiple groups were made by one-way ANOVA, followed by the Tukey test for multiple comparisons. Statistical analysis was performed by the GraphPad Prism software (GraphPad Software Inc., La Jolla, CA). *P* values of less than 0.05 were considered statistically significant.

## 3. Results

### 3.1. General Characteristics

As shown in Table 1, at five weeks of diabetes, water intake and food consumption were significantly higher while body weight was significantly lower in diabetic rats than those in nondiabetic control. And blood glucose in the diabetic rats (31.5 ± 2.6 mM) was much higher than control rats. FoxO1 inhibitor AS did not significantly affect blood glucose, nor did it have significant impact on water intake, food consumption, and body weight. APN significantly reduced water intake and food consumption but had no significant effect on body weight.

### 3.2. FoxO1 Inhibition and APN Supplementation Both Reduce Hepatic Fatty Infiltration in NAFLD Rats

**Figure 1:**
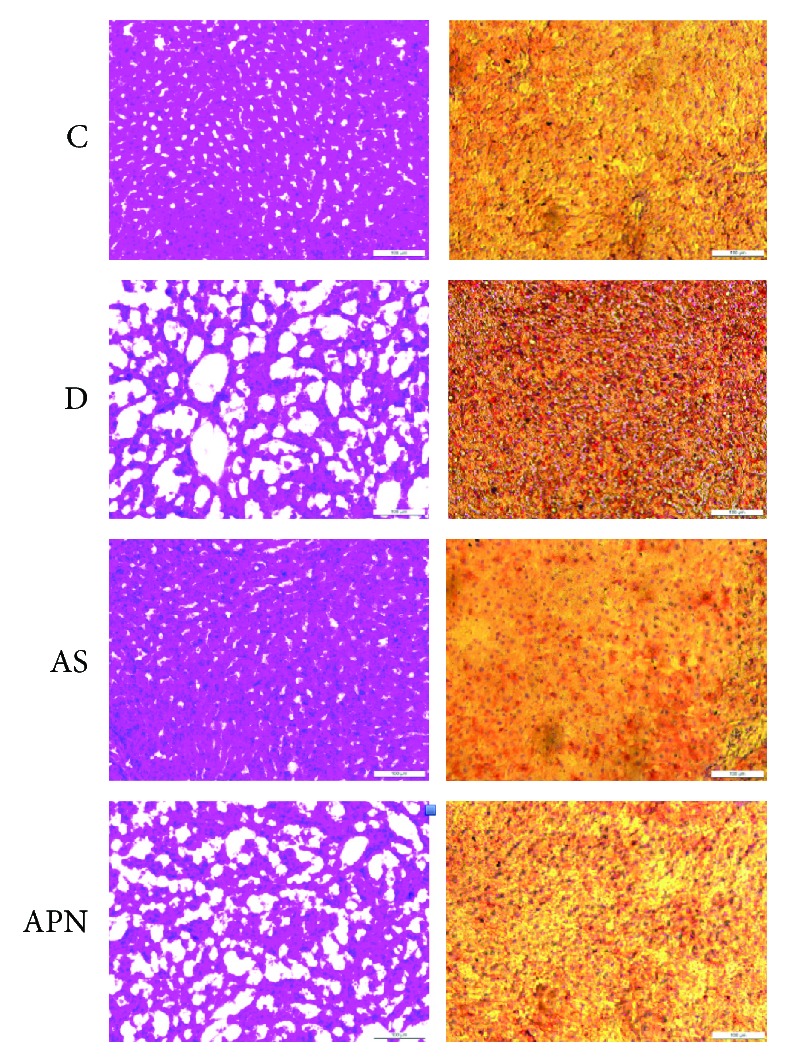
Representative H&E (hematoxylin and eosin) and Oil Red O staining of liver structures in different groups of rats. Images of staining under a microscope (magnification, ×20). C: control group; D: diabetic group; AS: AS-treatment group; APN: APN-treatment group.

As shown in Figure 1, under the microscope, the H&E and Oil Red O staining in the normal control group showed a clear structure, with little fat vacuoles. While in the diabetic control group, the liver structure was not clear and filled with different sizes of fat drops. In two treatment groups (especially the AS group), the liver showed an ameliorated structure and decreased fat drops.

### 3.3. FoxO1 Inhibition and APN Supplementation, Respectively, Ameliorated the Liver Function in T1DM-Induced NAFLD

**Figure 2:**
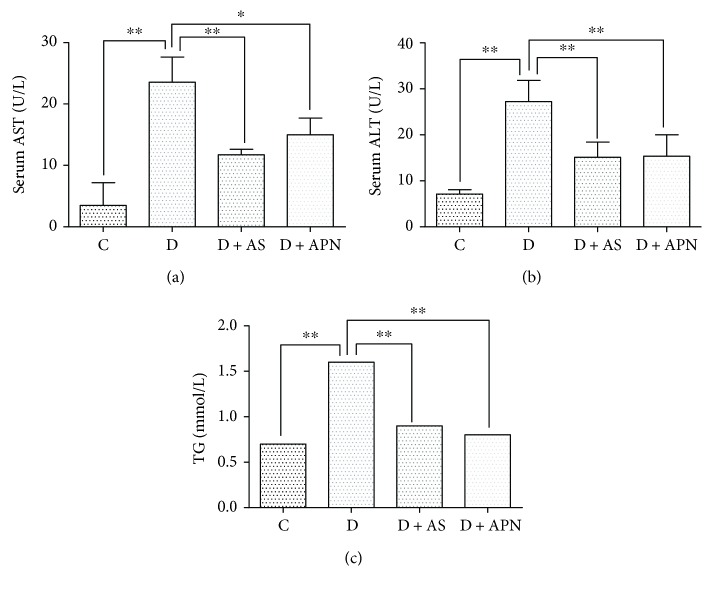
Effects of the FoxO1 inhibitor (AS) and APN on serum AST, ALT, and TG. Serum AST, ALT, and TG were determined by kits (Stanbio Laboratory, TX, USA). Values are expressed as mean ± SD, *n* = 6/group. ^∗^*P* < 0.05, ^∗∗^*P* < 0.01.

To investigate the effect of FoxO1 and APN on the liver function in T1DM-induced NAFLD, we first confirmed the effect of T1DM-induced NAFLD on the liver function. As shown in Figures 2(a)–2(c), serum AST, ALT, and TG were significantly increased in the diabetic group as compared with the control group. To explore the molecular mechanisms underlying the increase of serum AST, ALT, and TG, we examined the involvement of FoxO1, which is the key intracellular target of insulin effect and contributes to the regulation of lipid and glucose metabolism [14]. We found that the inhibition of FoxO1 through its selective inhibitor AS significantly decreased the serum AST, ALT, and TG compared with the solvent control, indicating the amelioration of the liver function. Also, the serum AST, ALT, and TG were apparently decreased under the treatment of APN, an antidiabetic and anti-inflammatory adipokine. Collectively, these results show that both AS and APN treatment improved the liver function in T1DM-induced NAFLD.

### 3.4. APN but Not the FoxO1 Inhibitor AS Reduced Blood Glucose in T1DM-Induced NAFLD Rats

**Figure 3:**
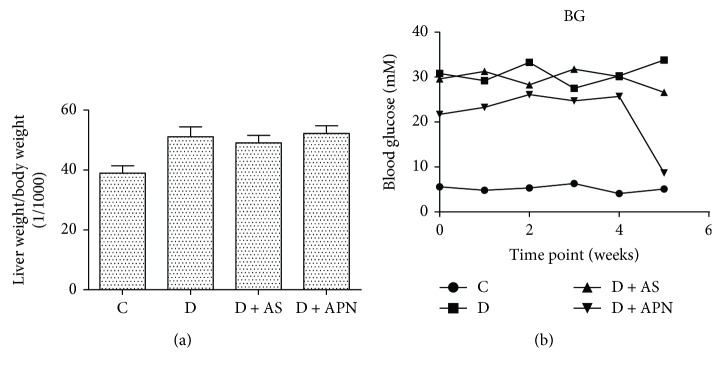
Effects of AS and APN on liver weight/body weight and weekly blood glucose. Blood glucose was measured by the OneTouch Ultra glucose meter. Values are expressed as mean ± SD, *n* = 6/group.

We next examined the effect of AS and APN on liver weight and blood glucose in T1DM-induced NAFLD. The ratio of liver weight/body weight in the diabetic rats was about 25% higher than that in the nondiabetic rats, although the difference did not reach statistical significance. Treatment with AS or APN did not have significant influence on the ratio of liver weight/body weight (Figure 3(a)). APN but not AS significantly reduced the blood glucose level (Figure 3(b)).

### 3.5. FoxO1 Inhibition and APN Supplementation, Respectively, Decreased Liver Nuclear FoxO1 in T1DM-Induced NAFLD

**Figure 4:**
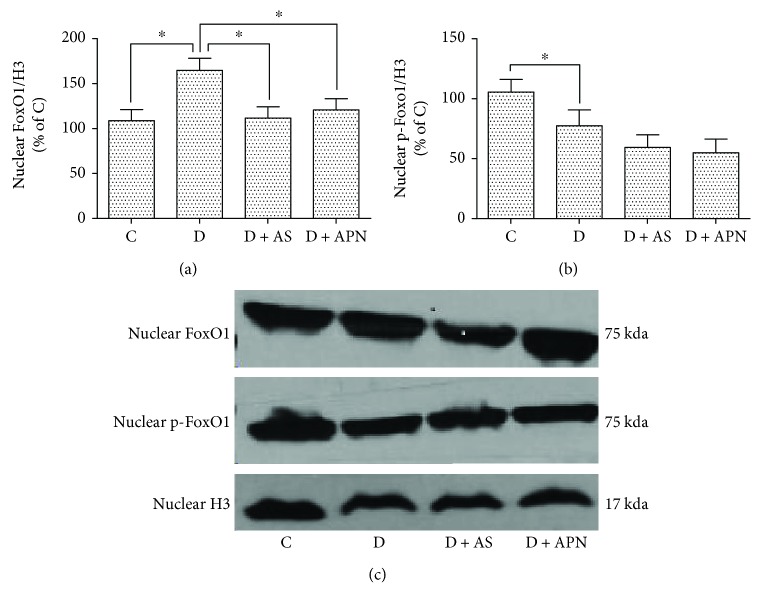
Effects of AS and APN on the expression of FoxO1 and p-FoxO1 in nucleus in the liver. Values are expressed as mean ± SD, *n* = 6/group. ^∗^*P* < 0.05.

Akt phosphorylates and excludes FoxO1 from the nucleus, retaining it to cytoplasmic compartment, thereby dampening its activity to produce glucose [33]. As shown in Figures 4(a)–4(c), the diabetic group demonstrated significant increase of nuclear FoxO1 expression. In order to further investigate how APN and AS ameliorate liver function in T1DM-induced NAFLD, we examined the effect of APN and AS on nuclear FoxO1, and the data showed that both APN and AS significantly decreased the expression of nuclear FoxO1 compared with control. AS and APN also tended to reduce nuclear p-FoxO1, but the difference did not research statistical significance.

### 3.6. Inhibition of FoxO1 Had No Significant Impact on the Expression of APN and AdipoR1 in T1DM-Induced NAFLD

APN is an antidiabetic adipokine which is reported to decrease after 7 weeks of T1DM induction [34], which is in accordance with our data shown in Figure 4(b). With regard to the AdipoR, we found that AdipoR1 is apparently increased in the diabetic group compared with the control group (Figure 4(a)), while application of AS did not show significant influence on the expression of APN and AdipoR1. In addition, AdipoR2 expression did not show significant difference in all groups (data not shown).

### 3.7. Liver Akt1 Was Significantly Reduced in Diabetic Rats and APN Restored Akt1 While AS Attenuated Reduction of Akt1 Expression

**Figure 5:**
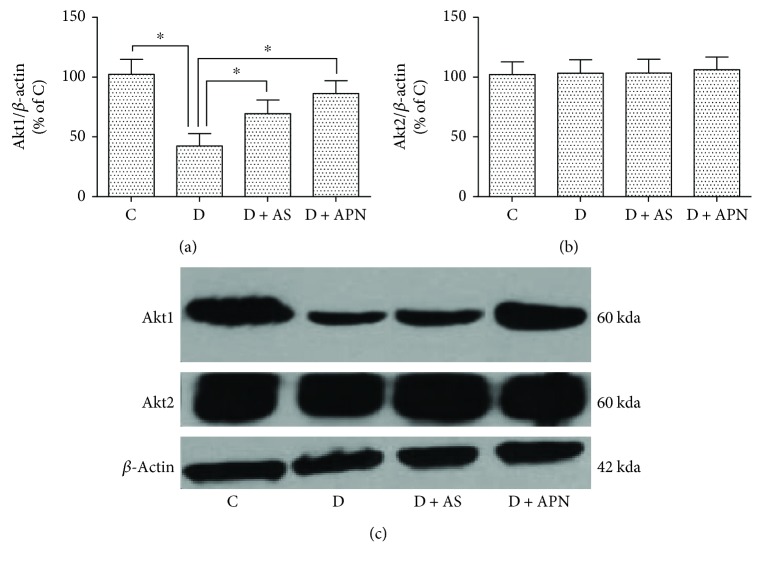
Effects of AS and APN on the expression of Akt1 and Akt2 in the liver. Values are expressed as mean ± SD, *n* = 6/group. ^∗^*P* < 0.05.

Since both APN and AS could decrease the expression of FoxO1, we further examined two subtypes of Akt, which is illustrated to function upstream of FoxO1 [35]. As shown in Figure 5, Akt1 expression was significantly reduced in the diabetic group. Application of APN restored Akt1 expression while AS moderately but significantly increased Akt1 expression. By contrast, the expression of Akt2 did not display apparent difference among all the groups.

### 3.8. Liver SIRT1 and Acetyl-FoxO1 Were Significantly Reduced in Diabetic Rats and Were Not Affected by Neither APN Nor AS

**Figure 6:**
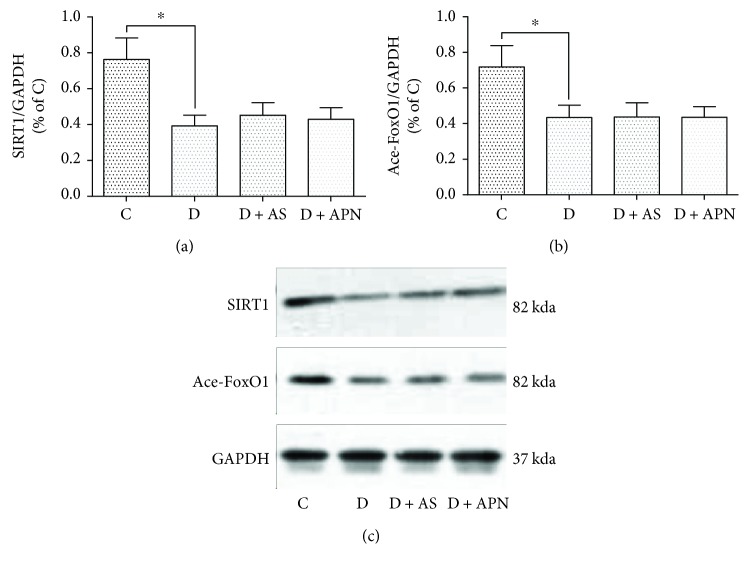
Effects of AS and APN on the expression of SIRT1 and acetyl-FoxO1 (Ace-FoxO1) in the liver. ^∗^*P* < 0.05.

It has been suggested that SIRT1 was supposed to function as an upstream regulator of FoxO1 actions in the liver and other tissues and that SIRT1 usually deacetylates FoxO1 and not necessarily change the FoxO1 protein levels. We, therefore, further measured the levels of liver SIRT1 and acetyl-FoxO1. As shown in Figures 6(a)–6(c), the liver SIRT1 and acetyl-FoxO1 were significantly decreased in diabetes, while AS and APN did not have significant impact on the reductions of SIRT1 and acetyl-FoxO1 (all *P* > 0.05).

## 4. Discussion

As one of the worldwide metabolic diseases, NAFLD represents a disease spectrum which ranges from simple steatosis to NASH, cirrhosis to end-stage carcinoma. It has been long considered that fatty liver is closely linked with T2DM, while the association between NAFLD and T1DM lacks enough attention. Although a few studies reported that the NAFLD is linked with T1DM [5, 6], the underlying mechanism is still unknown and needs further exploration.

**Figure 7:**
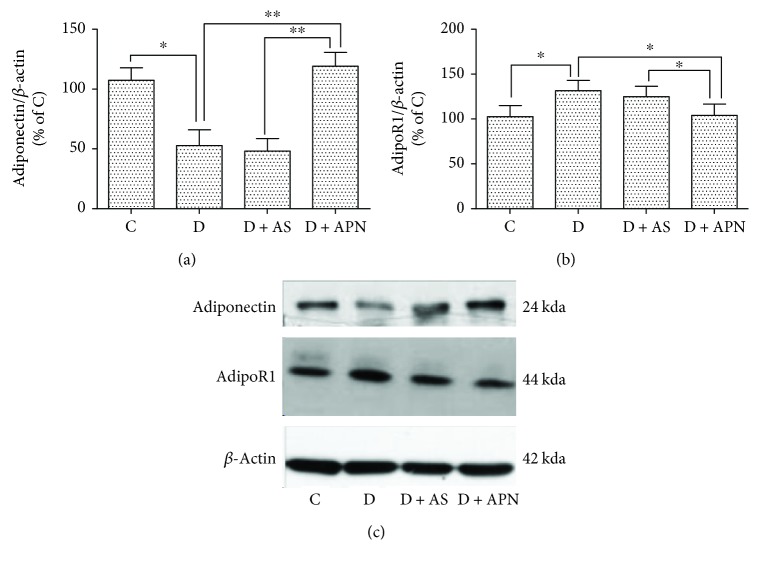
Effects of AS and APN on the expression of plasma adiponectin and liver adiponectin receptor 1 (AdipoR1) in diabetic rats. ^∗^*P* < 0.05, ^∗∗^*P* < 0.01.

APN is a multipurpose adipokine which is primarily secreted in adipose tissues and then released into the blood, further exerting antidiabetic and anti-inflammatory properties. In accordance with a previous study [36], we confirmed that APN significantly decreases blood glucose compared with the diabetic group, whereas AS had no obvious effect on blood glucose (Figure 3(b)). In addition, APN was reported to block cannabinoid type 1 receptor [37] and increase free fatty acid *β*-oxidation [38], therefore ameliorating the fat accumulation in the liver. In another study, the level of APN was found to be negatively related to the progression of NAFLD and may be a potential marker to distinguish different stages of NAFLD [9]. In this study, the serum APN level and hepatic APN expression were also significantly low in the diabetic group (Figure 7(a) and Table 1). However, the underlying molecular mechanism is still largely unknown. In the present study, we have demonstrated beneficial effects of APN on T1DM-induced NAFLD and explored its molecular mechanisms. We demonstrated that APN inhibited T1DM-induced NAFLD via reduction of the expression of FoxO1. Moreover, Akt1 signaling may represent a mechanism by which APN mediated the suppression of FoxO1 in T1DM-induced NAFLD (Figure 7).

FoxO1 is a commonly expressed member of the forkhead factor family and has long been considered to be involved in metabolic disorders, especially the lipid and glucose metabolism. On the contrary, blockade of FoxO1 increases lipogenesis and helps to restore metabolism homeostasis [14, 18, 19]. In the present study, we block the FoxO1 with selective antagonist (AS1842856) to examine its effect on T1DM-induced NAFLD. The data showed that liver function is impaired under diabetic condition, while the application of AS ameliorates liver function manifested as the reduction of ALT and AST (Figure 2). Interestingly, application of APN showed similar amelioration of liver function (Figure 2). Additionally, AS decreased the expression of nuclear FoxO1 and so did the APN (Figure 4(a)). Previous study reported that AS could change blood glucose [20]; however, in our study, blood glucose in the AS group showed no significant difference compared with the control group. We argue that this is mainly because of the different operations on the animals, since in that study mice were orally administered AS1842856 at three time points on the second day and went through fasting whereas our rats are administrated 2 times a day with a 12-hour interval for a duration of 8 days, which may have a different effect on the blood glucose. Considering that AS did not influence the level of APN (Figure 7(a)), we propose that APN is on the upstream of FoxO1 signal pathway. To confirm this hypothesis, we examined two subunits of Akt (Akt1 and Akt2). The result indicated that APN could restore the expression of Akt1 which is impaired in the diabetic group (Figure 5(a)), while Akt2 expression showed no significant difference (Figure 5(b)).

In summary, in this study, we examined the expression levels of APN and FoxO1 to explore the potential role of these two proteins in the pathology of type 1 diabetes-induced NAFLD. Findings obtained from the current study suggest that APN ameliorates NAFLD via inhibition of FoxO1 through Akt1/FoxO1 signaling pathway.

## Figures and Tables

**Table 1:** General characteristics.

Parameters	C	D	D + AS	D + APN
Water intake (mL/kg/day)	118.1 ± 13.7	898.3 ± 74.9^∗∗^	933.7 ± 161^∗∗^	475.6 ± 91.9^∗∗^^#^
Food consumption (g/kg/day)	77.5 ± 12.2	186.8 ± 13.7	168.7 ± 22^∗∗^	127.6 ± 18.2^∗∗^^#^
Body weight (g)	455.8 ± 46.9	294 ± 23.6^∗∗^	276 ± 26^∗∗^	267 ± 24.9^∗∗^
Serum insulin (ng/mL)	5.7 ± 0.9	1.2 ± 0.5	2.1 ± 0.6	4.9 ± 0.8^#^
Plasma APN levels (mg/mL)	16.3 ± 2.5	10.2 ± 1.7	11.5 ± 2.4	18.5 ± 2.7^#^

Data are mean ± SEM, ^∗∗^*P* < 0.01 versus C; ^#^*P* < 0.05 versus D.

## Data Availability

The data used to support the findings of this study are available from the corresponding author upon request.
